# Prevalence of thyroid dysfunction among Iranian older adults: a cross-sectional study

**DOI:** 10.1038/s41598-023-49085-2

**Published:** 2023-12-08

**Authors:** Maryam Mirahmad, Asieh Mansour, Mitra Moodi, Elaheh Safkhani, Vahid Haghpanah, Pooria Asili, Hossein Fakhrzadeh, Moloud Payab, Mahbube Ebrahimpur, Masoumeh Khorashadi, Huriye Khodabakhshi, Ali Akbar Esmaeili, Gholamreza Sharifzadeh, Asghar Zarban, Farshad Sharifi, Sayed Mahmoud Sajjadi-Jazi

**Affiliations:** 1 Endocrinology and Metabolism Research Center, Endocrinology and Metabolism Clinical Sciences Institute, Tehran University of Medical Sciences, Tehran, Iran; 2 Social Determinants of Health Research Center, Birjand University of Medical Sciences, Birjand, Iran; 3 Department of Internal Medicine, Shariati Hospital, Tehran University of Medical Sciences, Tehran, Iran; 4 Elderly Health Research Center, Endocrinology and Metabolism Population Sciences Institute, Tehran University of Medical Sciences, Tehran, Iran; 5 Non-Comunicable Disease Research Center, Endocrinology and Metabolism Population Sciences Institute, Tehran University of Medical Sciences, Tehran, Iran; 6 Department of Gerontology, University of Social Welfare and Rehabilitation Sciences, Tehran, Iran; 7 Iranian Research Center of Aging, University of Social Welfare and Rehabilitation Sciences, Tehran, Iran; 8 Department of Psychiatry, School of Medicine Medical Toxicology & Drug Abuse Research Center, Birjand University of Medical Sciences, Birjand, Iran; 9 Department of Epidemiology and Biostatistics, School of Health, Social Determinants of Health Research Center, Birjand University of Medical Sciences, Birjand, Iran; 10 Department of Clinical Biochemistry, School of Medicine, Cardiovascular Diseases Research Center, Birjand University of Medical Sciences, Birjand, Iran

**Keywords:** Endocrine system and metabolic diseases, Diseases, Endocrinology

## Abstract

There is limited data on the prevalence of thyroid dysfunction in the older population. This study aimed to determine the prevalence of thyroid dysfunction among a sample of Iranian older adults. A cross-sectional analysis of older adults who aged 60 years and over was conducted. A total of 363 subjects were randomly selected from Birjand longitudinal aging study (BLAS) cohort study. Serum thyroid-stimulating hormone (TSH) level, total thyroxine (T4) and total triiodothyronine (T3) were measured by the enzyme-linked immunosorbent assay (ELISA). Based on thyroid function tests and history of taking medicines used to treat thyroid disorders, participants were classified into the following groups: euthyroid, overt/subclinical hypothyroidism, and overt/subclinical hyperthyroidism. Subsequently, the crude and World Health Organization (WHO) age-standardized prevalence were estimated for different thyroid function categories. A total of 171 men and 192 women, aged 60–94 years, were randomly selected. The crude prevalence of total hypothyroidism was 22.31% (subclinical [18.46%], overt [3.86%]), and that of hyperthyroidism was 1.66% (subclinical [1.38%], overt [0.28%]). The crude prevalence of total thyroid dysfunction was, therefore, 23.97%. A female preponderance was noticed in both total (P-value = 0.035) and overt (P-value = 0.035) hypothyroidism. An increasing trend with age was noticed in the prevalence of total hypothyroidism (P-value = 0.049). Age-standardized prevalence of total hypothyroidism and hyperthyroidism was 26.63% (95% confidence interval [CI] 20.58–33.69%) and 1.11% (95% CI 0.49–2.51%), respectively. A considerable proportion of our study population demonstrated evidence of thyroid dysfunction, particularly subclinical hypothyroidism. Our findings highlight the importance of further investigation of thyroid disorders among older Iranian adults.

## Introduction

In the last few decades, life expectancy has steadily increased and the global population has aged at a rapid rate^1^. The increase in the elderly population is accompanied by increased rate of morbidity, with thyroid disorders being among the most prevalent common comorbidities^2^. The diagnosis of thyroid disorders in older adults is impeded by various factors, including comorbidities, physiological alterations in the hypothalamic-pituitary-thyroid axis, declined multiple organ functions, and superimposition of geriatric syndromes^3^.

Estimates of the prevalence of thyroid dysfunction are commonly based on findings from middle-aged populations^4,5^. In the general population, the prevalence of clinical hypothyroidism and hyperthyroidism are estimated to be about 0.2–5.3% and 0.8–1.3%, respectively^6^. However, the prevalence rate of thyroid dysfunction widely differs by several factors like gender, age, geographic areas, iodine status, and race/ethnicity of populations^7^. Previous studies reported that as the population age, the prevalence of thyroid disorders increased, mainly due to the increase in subclinical hypo- and hyperthyroidism^8,9^.

There is limited data regarding the prevalence of thyroid dysfunction in Iranian older adults. In light of the above, the aim of current research is to determine the prevalence of thyroid dysfunction among a sample of Iranian older adults.

## Materials and methods

### Study population

The study population comes from Birjand longitudinal aging study (BLAS). The protocol of the BLAS study is described elsewhere^10^. The BLAS is an ongoing prospective cohort study conducted on individuals aged ≥ 60 years, who were residents in the rural and urban areas of Birjand County. We performed a cross-sectional analysis of baseline data from urban representative sample of the BLAS study. Baseline data from the BLAS study were collected from September 2018 to July 2019. Included participants in the current study were selected based on random sampling from baseline of the BLAS study. The exclusion criteria was inability to refer to the laboratory center.

### Thyroid function tests

Overnight fasting blood samples were collected and centrifuged. The serum samples were stored at − 80 °C until biochemistry examinations.

Serum concentrations of thyroid-stimulating hormone (TSH), total thyroxine (T4) and total triiodothyronine (T3) were measured by the enzyme-linked immunosorbent assay (ELISA) (Padtan Gostar Isar, Iran) using a microplate reader machine (BioTek, USA). The lower limit of detection for TSH was 0.1 μIU/mL. The lower limit of detection for T4 and T3 was 1 mcg/dL and 20 ng/dL, respectively. The normal reference ranges for TSH, T4 and T3 were set at 0.45–4.49 μIU/mL, 4.5–12.6 mcg/dL and 80–220 ng/dL, respectively.

The thyroid status of participants was classified into one of the following five groups based on their thyroid parameters or current history of taking medicines used to treat thyroid disorders. Euthyroidism was defined as TSH level of 0.45–4.49 µIU/mL. Subclinical hypothyroidism was defined as TSH of 4.5–20 µIU/mL with T4 level within the reference range (i.e. 4.5 ≤ T4 ≤ 12.6 mcg/dL). Included participants were categorized as overt hypothyroidism if TSH level ≥ 20 µIU/mL, or TSH level ≥ 4.5 µIU/mL in combination with T4 level below the reference range, or previous history of overt hypothyroidism taking levothyroxine. A combination of TSH level less than 0.45 µIU/mL and T4 level within the reference range was defined as subclinical hyperthyroidism. Overt hyperthyroidism was defined as a serum TSH < 0.45 µIU/mL in combination with T4 > 12.6 mcg/dL or T3 > 220 ng/dL, or known case of overt hyperthyroidism taking propylthiouracil (PTU) or methimazole.

### Ethical consideration

The BLAS was approved by both the ethics committee of Endocrinology and Metabolism Research Institute of Tehran University of Medical Sciences (IR.TUMS.EMRI.REC.1396.00158) and Birjand University of Medical Sciences (IR.BUMS.Rec.1397.282). The research protocol of current study was also approved by the ethics committees of Tehran University of Medical Sciences (IR.TUMS.MEDICINE.REC.1400.504). Informed consent was obtained from all participants.

### Statistical analysis

We calculated the crude prevalence of different thyroid function categories according to sex and age groups. Moreover, we weighted the crude prevalence according to the weight of sex-age groups of the Birjand population. Then, World Health Organization (WHO) standard population was applied for estimation of age-standardized prevalence of thyroid disorders. Categorical variables were presented as frequency and percentage values. Continuous variables were presented as means ± standard deviations (SD). Each thyroid dysfunction category was compared to the euthyroid group using the chi-square test or Fisher's exact tests. All statistical analyses were performed using Stata software version 12 (Texas, USA). Statistical significance was determined to be at P-value ≤ 0.05.

## Results

A sample of 363 (192 females, 171 males) individuals were monitored for thyroid function. The mean age ± SD of the study participants was 69.34 ± 7.28, ranging from 60 to 94 years. The mean age of females and males was 68.70 ± 7.08 and 70.05 ± 7.45, respectively.

As shown in Table 1, the crude prevalence of total thyroid dysfunction was 23.97%. Of these, 22.31% of older adults were classified as hypothyroidism and 1.66% as hyperthyroidism.

**Table 1:** Crude prevalence of thyroid dysfunction in older adults classified according to sex.

Diagnosis	Total (n = 363)		Female (n = 192)		Male (n = 171)		P-value^¥^
	n	%	n	%	n	%	
Euthyroid	276	76.03	137	71.35	139	81.29	Reference
Total thyroid dysfunction	87	23.97	55	28.65	32	18.71	**0.027**^**#**^
Hypothyroidism							
Subclinical	67	18.46	40	20.83	27	15.79	0.139^#^
Overt	14	3.86	11	5.73	3	1.75	**0.035**^**#**^
Total	81	22.31	51	26.56	30	17.54	**0.035**^**#**^
Hyperthyroidism							
Subclinical	5	1.38	3	1.56	2	1.17	0.684*
Overt	1	0.28	1	0.52	0	0.00	0.498*
Total	6	1.66	4	2.08	2	1.17	0.684*

Significant values are in bold.

^#^Chi-squared test.

*Fisher's exact test.

^**¥**^Thyroid dysfunction categories were compared to the euthyroid group.

We noticed a female preponderance (28.65% vs. 18.71%) in total thyroid dysfunction (P-value = 0.027). This female preponderance was significantly observed in both total (P-value = 0.035) and overt hypothyroidism (P-value = 0.035). However, no sex-related difference was spotted in the prevalence of overt or subclinical hyperthyroidism. Subclinical hypothyroidism was the most common thyroid dysfunction, which occurred in 67 subjects (18.46%).

We further calculated the age-standardized prevalence of thyroid disorders using WHO standard population (Table 2). The age-standardized prevalence of total thyroid dysfunction, hypothyroidism, and hyperthyroidism in males was 26.40% (95% confidence interval [CI] 18.48–36.20%), 25.55% (95% CI 17.73–35.33%), and 0.85% (95% CI 0.22–3.26%), respectively, whereas for females it was 30.17% (95% CI 21.81–40.08%), 28.77% (95% CI 20.54–38.70%), and 1.40% (95% CI 0.49–3.91%), respectively.

**Table 2:** WHO age-standardized prevalence of thyroid dysfunction in older adults classified according to sex.

Diagnosis	Total		Female		Male	
	Prevalence, %	95% CI	Prevalence, %	95% CI	Prevalence, %	95% CI
Euthyroid	72.26	65.19–78.38	69.83	59.92–78.19	73.60	63.80–81.52
Total thyroid dysfunction	27.74	21.62–34.81	30.17	21.81–40.08	26.40	18.48–36.20
Hypothyroidism						
Subclinical	21.58	16.18–28.16	22.41	15.40–31.42	22.05	14.51–32.04
Overt	5.05	2.29–10.78	6.36	2.55–14.98	3.49	0.88–12.91
Total	26.63	20.58–33.69	28.77	20.54–38.70	25.55	17.73–35.33
Hyperthyroidism						
Subclinical	0.97	0.39–2.37	1.13	0.34–3.73	0.85	0.22–3.26
Overt	0.14	0.02–1.00	0.27	0.04–1.87	No obs	No obs
Total	1.11	0.49–2.51	1.40	0.49–3.91	0.85	0.22–3.26

*Obs* observation.

Participants were further divided based on their ages into three groups of 60–69 years, 70–79 years, and ≥ 80 years. The crude age-specific prevalence of thyroid dysfunction in older adults is presented in Table 3. An increasing trend with age was noticed in the prevalence of subclinical (P-value = 0.035) and total hypothyroidism (P-value = 0.049).

**Table 3:** Crude prevalence of thyroid dysfunction in older adults classified according to age.

Diagnosis	Age (years)			P-value^¥^
	60–69 (n = 211)	70–79 (n = 118)	≥ 80 (n = 34)	
Euthyroid, n (%)	166 (78.67)	89 (75.42)	21 (61.76)	Reference
Total thyroid dysfunction, n (%)	45 (21.33)	29 (24.58)	13 (38.24)	0.099^#^
Hypothyroidism, n (%)				
Subclinical	31 (14.69)	25 (21.19)	11 (32.35)	**0.035**^**#**^
Overt	9 (4.27)	3 (2.54)	2 (5.88)	0.419*
Total	40 (18.96)	28 (23.73)	13 (38.24)	**0.049**^**#**^
Hyperthyroidism, n (%)				
Subclinical	4 (1.90)	1 (0.85)	0 (0.00)	0.771*
Overt	1 (0.47)	0 (0.00)	0 (0.00)	1.000*
Total	5 (2.37)	1 (0.85)	0 (0.00)	0.792*

Significant values are in bold.

^#^Chi-squared test.

*Fisher's exact test.

^**¥**^Thyroid dysfunction categories were compared to the euthyroid group.

Table 4 shows the WHO age-standardized prevalence of thyroid dysfunction categorized by age groups. The age-standardized prevalence of total thyroid dysfunction and total hypothyroidism were found to increase by age, with the highest prevalence found within the age group ≥ 80 years.

**Table 4:** WHO age-standardized prevalence of thyroid dysfunction in older adults stratified by age.

Diagnosis	Age-standardize prevalence, % (95% CI)		
	60–69 years	70–79 years	≥ 80 years
Euthyroid	79.45 (73.47–84.37)	74.93 (66.16–82.05)	61.85 (43.21–77.55)
Total thyroid dysfunction	20.55 (15.63–26.53)	25.07 (17.95–33.84)	38.15 (22.45–56.79)
Hypothyroidism			
Subclinical	14.13 (10.07–19.47)	21.48 (14.90–29.94)	29.62 (16.28–47.68)
Overt	4.02 (2.09–7.61)	2.74 (0.88–8.20)	8.52 (2.11–28.74)
Total	18.15 (13.54–23.89)	24.21 (17.22–32.92)	38.15 (22.45–56.79)
Hyperthyroidism			
Subclinical	1.99 (0.74–5.22)	0.85 (0.12–5.81)	No obs
Overt	0.41 (0.06–2.86)	No obs	No obs
Total	2.40 (1.00–5.66)	0.85 (0.12–5.81)	No obs

*Obs* observation.

## Discussion

This cross-sectional analysis aimed to identify the prevalence of thyroid dysfunction in older adults who live in Birjand, Iran. A considerable proportion of our study population (23.97%) demonstrated evidence of thyroid dysfunction, which remained notable in age-standardized model (27.74% [95% CI 21.62–34.81%]). This finding is in keeping with a previous study that reported the prevalence of thyroid dysfunction among older adults as about 25% in the US^11^. Results from Europe and Australia revealed a lower prevalence of total thyroid dysfunction. In Australia, a prevalence of 10% (95% CI 8.9–11.1%) was reported in older adults^12^. The mean prevalence of thyroid dysfunction in Europe, including different age groups, was 3.82% (95% CI 3.77–3.86%)^4^.

We estimated the age-adjusted prevalence of hyperthyroidism as about 1.11% (subclinical [0.97%] and overt [0.14%]). Several studies have been conducted to investigate the prevalence of hyperthyroidism. In the general population, the prevalence of overt hyperthyroidism is estimated to be about 0.2–1.3% in iodine-sufficient areas^4,13,14^. In a meta-analysis conducted on European populations, the prevalence of subclinical hyperthyroidism was calculated as 2.91% (95% CI 2.63–3.21%)^4^. The prevalence of subclinical and overt hyperthyroidism in a population of Mexican older adults was 0.5% (95% CI 0.18–0.88) and 0.6% (95% CI 0.22–0.95), respectively^15^.

The age-standardized prevalence of hypothyroidism in the present study was estimated to be 26.63% (95% CI 20.58–33.69%), with a clear dominance of subclinical types of the disorder: subclinical hypothyroidism 21.58% (95% CI 16.18–28.16%) and overt hypothyroidism 5.05% (95% CI 2.29–10.78%).

The global prevalence of overt hypothyroidism is about 0.2–5.3%, and the prevalence of subclinical hypothyroidism is about 4–15%^6,16,17^. The prevalence of hypothyroidism can be affected by a number of factors like age, gender, ethnicity, and iodine status^18^.

Based on the results from a meta-analysis of the European population, a higher prevalence of hypothyroidism was found in females and those aged ≥ 65 years^18^. In the US, hypothyroidism occurs in about 21% of women aged 75 years and older^5^. Our results regarding female preponderance in thyroid dysfunction are in line with previous findings^2,4,5^.

In subjects over 65 years, the prevalence of subclinical hypothyroidism was reported as about 17.3% in a Korean study^19^, and about 6.5% (95% CI 5.2–7.8%) in a Brazilian study^20^. Juárez-Cedillo et al. have reported that subclinical hypothyroidism occurs in about 15.4% (95% CI 13.40–16.81%) of Mexican older adults, with overt hypothyroidism occurring in about 7.5% (95% CI 5.91–8.36%)^15^. Moreover, it was previously shown that about 20% of community-dwelling subjects aged 65 years and over were taking a thyroid hormone preparation^2^.

Similar to previous studies, our study also showed that subclinical hypothyroidism is the most common form of thyroid disorder in the elderly. Subclinical hypothyroidism is not exclusive to the elderly. However, given the data indicating the rise in TSH levels with age, it is crucial to determine whether this phenomenon is detrimental, beneficial or neutral in the elderly. Although a higher TSH level might be "normal" in older people, we still need to recognize the impact of the slightly decreased thyroid hormone levels on the population as they age^21^. Many subjects with subclinical hypothyroidism are asymptomatic and older populations seem to experience fewer symptoms than younger populations^22–25^. Notably, one study on participants aged 70–79 years, reported a higher proportion of subjects with good cardiorespiratory fitness and better mobility in participants with mild subclinical hypothyroidism than the age- and sex-adjusted euthyroid group^23^. Nevertheless, a more recent study did not confirm these findings^26^. It was also shown that treatment of subclinical hypothyroidism with levothyroxine in people younger than 70 years improved risk of ischemic heart disease, whereas this was not evident in people older than 70 years old^27^. The risk of mortality is increased in both under- and over-treatment hypothyroidism, with higher risks with over-treatment^28^. Based on this evidence, many experts suggest higher cut-off values of TSH levels for treatment in older populations^25,29,30^.

Differences in the prevalence of thyroid disorders among different populations might be attributed to the differences in iodine intake levels. Iodine status is known to be a major factor affecting the prevalence of thyroid disorders in older adults^3^. Both iodine excess and deficiency can lead to hyperthyroidism. Toxic nodular goiter is the most common cause of hyperthyroidism in the elderly, particularly in the areas with iodine-deficiency^14,31^. In addition, hypothyroidism is more prevalent in both populations with high iodine intake and those with moderate to severe iodine-deficiency. The iodine consumption of people should ideally be kept within a narrow range, where disorders related to deficiency of iodine are prevented, but not higher^32^. In 1968, for the first time, goiter was identified as an endemic condition in different regions of Iran^33^. In order to control iodine deficiency disorders in Iran, iodine supplementation programs were started in 1989, and mandatory iodized salt consumption became law since 1994^34,35^. Although Iran is currently considered an iodine-sufficient country, salt consumed in some provinces of Iran demonstrated moderate levels of iodine deficiency^36^. According to a survey performed to determine urinary iodine status in Iranian students aged 8–10 years old, Southern Khorasan province, where Birjand is located, was among the provinces with the lowest mean urinary iodine excretion (11.8 μg/dL)^37^.

The present study has some limitations. Considering the singleton measurement of thyroid function tests, the possibility of transient changes in some thyroid function tests cannot be ruled out. Moreover, we did not measure serum free thyroid hormones and anti-TPO levels. Finally, studies with larger sample sizes are required to confirm the present observations.

## Conclusion

Our study showed a high proportion of Iranian older adults have thyroid dysfunction, particularly subclinical hypothyroidism. This high prevalence sheds light on the importance of further investigation of thyroid dysfunction because of adverse health outcomes of thyroid disorders in this age group with other complex comorbidities.

## Data Availability

Data described in the manuscript, code book, and analytic code will be made available upon reasonable request from the corresponding author.
